# Characterization of Lactic Acid Bacteria Isolated From Rotting Oranges and Use of Agropastoral Processing By-products as Carbon and Nitrogen Sources Alternative for Lactic Acid Production

**DOI:** 10.1155/2024/4264229

**Published:** 2024-09-09

**Authors:** Romial Joel Ngouénam, Ghadir Nofal, Sanjukta Patra, Bilkissou Njapndounke, Edith Marius Foko Kouam, Pierre Marie Kaktcham, François Zambou Ngoufack

**Affiliations:** ^1^ Laboratory of Microbiology Department of Microbiology Faculty of Science University of Yaoundé I, PO Box 812, Yaoundé, Cameroon; ^2^ Enzyme and Microbial Technology Laboratory Department of Biosciences and Bioengineering Indian Institute of Technology Guwahati 781039, Guwahati, Assam, India; ^3^ Department of Physiological Sciences and Biochemistry Faculty of Medicine and Pharmaceutical Sciences University of Dschang, Dschang, Cameroon; ^4^ Research Unit of Biochemistry Medicinal Plants Food Science and Nutrition (URBPMAN) Department of Biochemistry Faculty of Science University of Dschang, PO Box 67, Dschang, Cameroon

**Keywords:** chicken by-products, fish by-products, lactic acid, *Lactiplantibacillus plantarum*, lyophilization, papaya by-products, yeast extract

## Abstract

This study investigated the ability of lactic acid bacteria (LAB) isolated from oranges to use fish by-products (FB) and chicken by-products (CB) as nitrogen sources alternative to yeast extract for lactic acid (LA) production in a papaya by-product medium as a carbon source. Once the fermentation agents had been isolated, they were subjected to biochemical and molecular characterization. Inexpensive nitrogen sources, precisely CB and FB, were prepared, freeze-dried, and yield evaluated. Also, before to the fermentation experiments, the Total Kjehdahl Nitrogen (TKN) of these by-products and that of the yeast extract were determined. Then, three production media differing in terms of nitrogen source were formulated from these nitrogen sources. From the 22 LAB isolated from orange, two isolates of interest (NGO25 and NGO23) were obtained; all belonging to the *Lactiplantibacillus plantarum* species based on 16S rRNA gene sequencing. Furthermore, the production yield powder obtained after lyophilization of 1 L of CB and FB surpernatant were, respectively, 16.6 g and 12.933 g. The TKN of different nitrogen sources powder were 71.4 ± 0.000% DM (FB), 86.145 ± 0.001% DM (CB), and 87.5 ± 0.99% DM (yeast extract). The best kinetic parameters of LA production (LA (g/L): 31.945 ± 0.078; volumetric productivity (g/L.h): 1.331 ± 0.003; LA yield (mg/g) 63.89 ± 0.156; biomass (g/L) 7.925 ± 0.035; cell growth rate (g/L.h): 0.330 ± 0.001) were recorded by *Lactiplantibacillus plantarum* NGO25 after 24 h of fermentation. The latter data were obtained in the production medium containing CB as nitrogen sources. In addition, this production medium cost only $0.152 to formulate, compared to yeast extract which required $1.692 to formulate. Thus, freeze-dried CB can be used as an alternative to yeast extract in large-scale production of LA.

## 1. Introduction

Reducing the production costs of a molecule of economic interest enables the industry to make considerable profits. This ideology involves the search for inexpensive-good quality raw materials [1]; the latter is the aim of authors who have been interested in the production of LA for decades. LA is an organic compound that exists in two isomers (D and L) [2]; due to its environmental friendliness and purity of the end product (optically pure D- or L-LA that are subject to biodegradable polymers production), it is produced preferentially by fermentation using bacteria and fungi as fermentation agents [3–5]. Due to its various applications (cosmetic, pharmaceutical, chemical, and food industries), the global LA market is growing. The latter would represent a turnover of $9.8 billion in 2025 for approximately 1960.1 kt produced [6]. However, glucose, sucrose, lactose, maltose, yeast extract, ammonium sulfate, urea, and peptone are extremely expensive substrates which are among the main problems to the production cost of LA by fermentative pathway [7, 8]. Indeed, the literature highlights a large number of scientific works [9–14] that have tried to solve this cost problem by promoting the use of by-products of agropastoral processing (paper sludge, whey powder, sugarcane bagasse, rice bran, groundnut oil cake, wheat bran, coconut oil cake, tea waste, sapota peel, corn cob powder, molasses, potato residue, banana peel, date waste, pineapple by-products, microalgal biomass of *Chlorella vulgaris*, cashew apple juice, rice straw, wastewater sludge, red seaweed hydrolysate, lucerne green juice, peanut meal, soya okara hydrolysate, wheat straw, dregs, chicken feather hydrolysate, ram horn, corn steep liquor, oat groats, fish waste, and food waste) as inexpensive carbon and nitrogen sources for LA production. In the same token, Ngouénam et al. [15], in their studies, used papaya by-products as carbon source for lactic acid production by *Lactiplantibacillus plantarum* strains isolated from tropical fruits. These works also demonstrated insufficient bacterial growth and consequent low LA production due to the low nitrogen content of papaya by-products. This observation highlighted the need to supplement this production medium with a nitrogen source. To address this need, these authors supplemented this production broth mainly with yeast extract, known as expensive and usually used for industrial production, thus needing to search for low-cost nitrogen source alternatives. In this sense, fish and chicken by-products (CB), given their nitrogen content [16, 17], could be potential sources of nitrogen used in LA production. In Cameroon, the fish and chicken processing and marketing chain is expanding rapidly and generating significant quantities of by-products with exploitable nitrogen content. In this regard, Ngouénam et al. [6] used fish and chicken by-products as a nitrogen source for LA production by the fermentative pathway. In addition, in their works the carbon source used was pineapple by-products, and the nitrogen sources (CB supernatant) used were not freeze-dried; however, lyophilization would help cancel the dilution effect of the production medium due to the addition of the supernatant, concentrate the nutrients further, and ease usage and storage.

Based on the above, the present research is aimed at characterizing LAB isolated from rotting oranges. Also, it investigates the challenge to increase LA productivity from papaya by-products, freeze-dried fish, and CB as alternative carbon and nitrogen sources.

## 2. Materials and Methods

**Figure 1:**
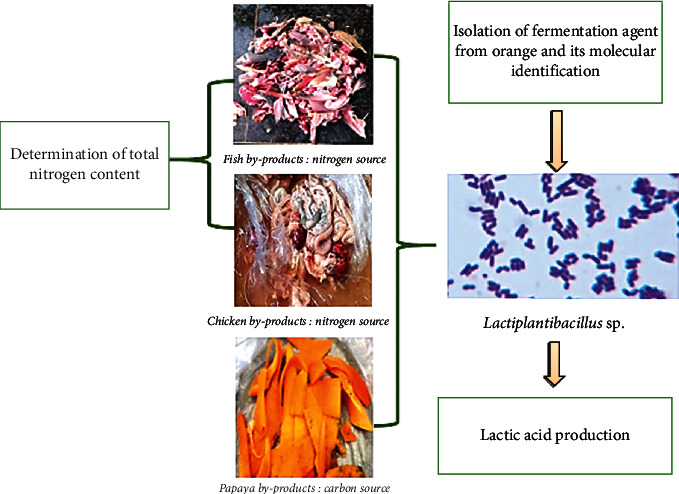
Graphical methodology.

The graphical methodology of the present investigation is illustrated in Figure 1.

### 2.1. Isolation and Characterization of Lactic Acid Bacteria (LAB)

#### 2.1.1. Isolation and Phenotypic and Biochemical Characterization of LAB

Overripe oranges from the main market of the city of Dschang (Menoua Division, West Cameroon region) were aseptically collected for isolation of Lactobacilli using MRS medium (TM MEDIA, Titan Biotech Ltd, India). All cultures were grown at 30°C under anaerobic conditions for 48 h, and pure LAB isolates were stored at −86°C in sterile MRS supplemented with glycerol (25%). The phenotypic, morphological, and biochemical characterization of pure LAB isolates was done as recommended by *Bergey's Manual of Systematic Bacteriology*. This included catalase activity, CO_2_ production from glucose, Gram staining, growth at 10°C and 45°C, NaCl tolerance (2, 4, 6.5%), indole test, methyl red test, Voges–Proskauer test, arginine hydrolysis test, gelatine hydrolysis test, urease test, and hydrogen sulfide production. In addition, field emission scanning electron microscopy (FESEM) was used to determine the macroscopic nature of the isolates of interest.

##### 2.1.1.1. Catalase Activity of Pure Isolates

According to the modified method of Sirisha, J. Lakshmi, and K. Lakshmi [18], catalase activity was tested on pure LAB isolates. On a microscope slide, a colony of the isolate was placed with the loop. Then, using a Pasteur pipette, few drops of 3% (*v*/*v*) hydrogen peroxide (H_2_O_2_) solution were placed on the colony. The release of oxygen marked by effervescence was considered a positive result. A strain of *Saccharomyces cerevisiae* isolate known for its production of catalase was used as a positive control.

##### 2.1.1.2. Gram Staining of LAB Isolates

Gram staining followed by microscopic observation was performed following the standard method to check the purity and the morphology of the isolates. A colony of the isolate from a 48-h culture on MRS-agar was plated on a slide, and the cells were fixed with heat. The fixed cells were stained with crystal violet and washed after 1 min with tap water. Then, the stained cells were treated with Lugol for 1 min and washed with tap water. Furthermore, the cells were washed with decolourizer for 15 s, stained with safranin for 30 s, and washed with tap water. After drying the slide, the cells were observed in a bright-field microscope under an oil immersion lens (100x). The Gram-negative bacteria were stained pink while the Gram-positive bacteria remained stained purple.

##### 2.1.1.3. CO_2_ Production of LAB Isolates From Glucose

A 0.5 mL volume of a pure Gram-positive, catalase-negative isolate reactivated for 24 h was inoculated into 2 mL of MRS agar plates maintained in supercooling (42°C–45°C). On top of this solidified inoculum, 2 mL of an agar-water mixture (1.5% w/v) was added, forming an “agar plug.” The prepared samples were incubated for 48 h at 30°C, and gas production was detected by displacement of the “agar plug.” Positive control was performed using *Saccharomyces cerevisiae* isolate known for its CO_2_ production [19].

##### 2.1.1.4. Growth of Isolates at 10°C and 45°C

It was assessed according to the protocol described by Bhardwaj et al. [20]. Briefly, 1% of the 24-h-old culture was inoculated into MRS broth medium, previously heated to the chosen temperature, and then incubated at 45°C or 15°C for 48 h and 10 days, respectively. Growth was assessed by examining the turbidity of the culture. A negative control consisting of a tube of uninoculated medium was incubated at the same temperatures and time.

##### 2.1.1.5. Effect of NaCl Concentrations on Growth of Isolates

The isolates were inoculated at the rate of 1% (*v*/*v*) in MRS broth having different NaCl concentrations (2.0%, 4.0%, and 6.5%) as described by Kavitha, Sindhuja, and Banumathi [21] and incubated at 30°C for 24–48 h. Growth was assessed by examining the turbidity of the culture.

##### 2.1.1.6. LAB Isolates Gelatinase Production

The modified method of Rohde et al. [22] was used to assess gelatinase production. Two microlitres (2 *μ*L) of culture from each isolate was deposited as a spot on the surface of nutrient agar containing gelatin (12%, w/v) and incubated at 30°C for 48 h. Following this incubation, the surface of the nutrient agar was flooded with a saturated solution of ammonium sulfate, and the clear areas around the LAB spots were searched for.

##### 2.1.1.7. Carbohydrate Utilization of Isolated LAB

The utilization of glucose, adonitol, arabinose, lactose, sorbitol, mannitol, and rhamnose, as carbon sources, was assessed using the KB001 HiIMViC Biochemical Test Kit (HIMEDIA). A single well-isolated colony of LAB was picked up and inoculated in 5 mL of nutrient broth and incubated at 30°C for 4–6 h until the inoculum turbidity was > 0.1 OD at 620 nm. The kit was aseptically opened, and each well was inoculated with 50 *μ*L of the above inoculum by surface inoculation method. After 24 h of incubation, the change in colour of the well from pinkish red/red to yellow indicated a positive result. Concerning fructose and sucrose, their utilization as a carbon source was assessed in MRS broth in which glucose was substituted by these different carbohydrates. Incubation conditions were as described above, and the culture tubes were observed for the presence or absence of growth.

##### 2.1.1.8. Arginine Hydrolysis Test

Based on the modified method of Bhardwaj et al. [20], the ability of LAB isolates to hydrolyze arginine was carried out. Autoclaved arginine dihydrolase broth tubes (5 mL) containing bromocresol purple (0.016 g/L) were inoculated with the pure culture (1%) and incubated at 30°C for 48 h. After incubation, the colour changed from purple to yellow, indicating a negative result for arginine hydrolysis; furthermore, the lack of change in the purple stain of the culture medium indicated a positive result.

##### 2.1.1.9. Hydrogen Sulfide Production by LAB Isolates

It was performed following the modified method described by Thakur, Anokhe, and Kalia [23]. Two microlitres (2 *μ*L) of culture from each LAB isolate was deposited as a spot on the surface of triple sugar-iron agar medium and incubated at 30°C for 48 h. After incubation, the black color of LAB spots indicates a positive result for hydrogen sulfide production.

##### 2.1.1.10. Indole, Methyl Red, and Voges–Proskauer Test

It was assessed using KB001 HIMEDIA (TM MEDIA, Titan Biotech Ltd, India). A single well-isolated colony of LAB was picked up and inoculated in 5 mL of nutrient broth and incubated at 30°C for 4–6 h until the inoculum turbidity was > 0.1 OD at 620 nm. The kit was aseptically opened, and each well was inoculated with 50 *μ*L of the above inoculum by surface inoculation method. After 24 h of incubation, the following operations were carried out:

1. 1–2 drops of Kovac's red reagent were added in the well, and the change in colour of the well from colourless to reddish pink indicated a positive result for the indole test.
2. 1–2 drops of methyl red reagent were added in the well, and the change in colour of the well from colourless/light yellow to red indicated a positive result for Methyl red test.
3. 1–2 drops of Baritt Reagent A and 1–2 drops of Baritt Reagent B were added in the well, and the change in colour of the well from colourless/light yellow to pinkish red indicated a positive result for Voges Proskauer's.

##### 2.1.1.11. Urease Production by Pure LAB

Following the protocol described by Benita [24], the production of urease by LAB isolates was assessed. A heavy inoculum from a 24-h pure culture was used to inoculate (1%) the urease test broth. Tubes were gently shaken to suspend the bacteria, and they were incubated at 30°C under agitation (180 rpm). The broth was observed for a color change at 8, 12, 24, and 48 h. Urease production was indicated by a bright pink (fuchsia) color throughout the broth.

##### 2.1.1.12. LAB Isolates Citrate Utilization

Using KB001 HIMEDIA (TM MEDIA, Titan Biotech Ltd, India), the capability of LAB to utilize citrate as a sole carbon source was performed. A single well-isolated colony of LAB was picked up and inoculated in 5 mL of nutrient broth and incubated at 30°C for 4–6 h until the inoculum turbidity was > 0.1 OD at 620 nm. The kit was aseptically opened, and each well was inoculated with 50 *μ*L of the above inoculum by surface inoculation method. After 24 h of incubation, the change in colour of the well from green to blue indicated a positive result.

##### 2.1.1.13. FESEM Analysis of Isolates of Interest

It was performed following the modified method of Chemana et al. [25] to determine the macroscopic nature of the isolates of interest. Ten milliliters of a 24-h culture (30°C, 180 rpm) was centrifuged (25°C/6000 rpm/7 min), and the pellets were collected and washed (25°C/6000 rpm/5 min) 3 times with PBS (pH 7.2) (1 mL); then, the supernatant was discarded. The pellet was resuspended in 1 mL of 0.25% glutaraldehyde/Na-phosphate (pH 7.2) and kept in the ice for 30 min followed by centrifugation (25°C/6000 rpm/5 min); the obtained pellet was resuspended in 1 mL of 0.25% glutaraldehyde/Na-phosphate (0.1 M, pH 7.2) and kept for overnight incubation (37°C, 180 rpm, 24 h). After overnight incubation, the sample was centrifuged (25°C/6000 rpm/5 min), the pellet collected was washed 3 times with Na-phosphate (pH 7.2), and the supernatant was discarded. The sample was dehydrated (25°C/6000 rpm/7 min) with different ethanol volumes (1 mL) as follows: 30%, 50%, 70%, 80%, 90%, and 100%; for each ethanol volume, the sample was incubated at room temperature for 10 min. Subsequently, the sample was incubated in 100% ethanol for 1 h and then centrifuged (25°C/6000 rpm/5 min); the micropipette was used to remove the supernatant. One hundred milliliters of 100% ethanol was added to the pellet to dissolve it, and then, 200 mL of this mixture was added in an Eppendorf containing 200 mL of distilled water. From the latter, 100 mL was collected and then diluted with 100 mL of 100% ethanol. Finally, the carbon tape was attached to the aluminum foil, and the sample (1.5 *μ*L) was deposited on the latter as a spot and incubated at 37°C for 24 h. After incubation, the sample was coated, and analysis was performed using Zeiss Gemini 300 FESEM.

#### 2.1.2. Molecular Identification of LAB Isolates by 16S rRNA Gene Sequencing

##### 2.1.2.1. Genomic DNA (GDNA) Extraction

CTAB-protocol described by Moore et al. [26] with modifications was used for GDNA extraction. Briefly, 15 mL of a 24 h culture (30°C, 180 rpm) was centrifuged (4°C/5800 rcf/7 min), and the pellets were collected and resuspended in 567 *μ*L TE (Tris-EDTA) buffer by repeated pipetting. Furthermore, 30 *μ*L of 10% SDS and 6 *μ*L of 10 mg/mL proteinase K were added to the test tube to give a final concentration of 100 *μ*g/mL proteinase K in 0.5% SDS; the whole contained in an Eppendorf tube (2 mL) was mixed thoroughly and incubated (37°C, 1 h 30 min,). Subsequently, 1 *μ*L of RNase was added to the test tube and incubated at room temperature for 15 min; after this incubation, 100 *μ*L of 5 M NaCl was introduced, and the suspension was mixed thoroughly and incubated at 65°C for 2 min. Eighty microliters of CTAB/NaCl solution (preheated at 65°C) was added to the previous suspension and thoroughly mixed and incubated for 10 min at 65°C. An approximately equal volume (0.7 to 0.8 mL) of chloroform/isoamyl alcohol solution was added to the Eppendorf tube, mixed thoroughly, and then centrifuged at 12000 RCF for 5 min. The upper (aqueous) phase (supernatant 1), containing the nucleic acids, was transferred into a separate 2 mL Eppendorf tube. The supernatant1 was extracted with an equal volume of phenol/chloroform/isoamyl alcohol solution, centrifuged 12,000 RCF for 5 min, and the upper (aqueous) phase (supernatant 2), containing the nucleic acids, was transferred into a separate 2 mL Eppendorf tube. To the latter was added 0.6 vol isopropanol to precipitate the nucleic acids, and the Eppendorf tube was shaken and force-stirred until a stringy white DNA precipitate becomes clearly visible. The mixture was centrifuged at 12,000 RCF for 10 min at room temperature (the DNA should be visible as a pellet on the side of the Eppendorf tube) and pellets (DNA) were collected. DNA was washed 3 times (with volumes of 400, 200, and 200 *μ*L of 70% ethanol, respectively) by centrifugation at 12,000 RCF for 2 min at room temperature to remove CTAB residues, and the supernatant was discarded. Subsequently, the opened tubes were centrifuged at 12000 RCF for 2 min and then dried in an oven (45°C, 5 min) and for 1 h under the laminar. Finally, pellets were redissolved in 30 *μ*L TE buffer and let sit at 37°C for 30 min to allow the DNA to be resuspended completely, and the concentration and purity of GDNA were estimated using a NanoDrop Spectrophotometer (Thermo Scientific, USA).

##### 2.1.2.2. PCR Amplification and Sequencing

PCR amplification was done using bacterial universal primers 27F and 1492R. The sequence of each primer is 5′-AGAGTTTGATCCTGGCTCAG-3′ and 5′-GGTTACCTTGTTACGACTT-3′, respectively [27]. Amplifications of 60 *μ*L were performed using 1.5 *μ*L of the GDNA solution, 1.2 *μ*L of each primer, 30 *μ*L of Master Mix, and 26.1 *μ*L of nuclease-free water. Amplifications were carried out in a thermal cycler (Agilent Technologies, SureCycler 8800, USA) according to the following program: initial denaturation at 95°C for 3 min, 30 cycles each with 30 s denaturation, 45 s annealing at 55.7°C (isolate NGO23) and 49.2°C (isolate NGO25), 1 min 24 s extension at 68°C, and final elongation step at 68°C for 10 min. During 1 h 30 min on a 0.8% agarose gel and electrophoresis at 90 V, the amplification products were separated. Ethidium bromide staining was used for the DNA band visualization. Using MACHEREY-NAGEL's clean-up kits, all fragments of about 1400 g bp (amplicons) on the agarose gel were purified and sent to commercial services of Eurofins India for sequencing. The 16S rRNA gene sequences of each isolate were compared with those in the NCBI GenBank database using the BLAST program (http://blast.ncbi.nlm.nih.gov/blast.cgi) for LAB identification. Finally, the sequences were deposited in the same database, and accession numbers were obtained.

### 2.2. Preparation of Powdered Nitrogen Sources From Chicken and Fish By-products (FB) and Determination of Nitrogen Content

#### 2.2.1. Nitrogen Source Preparation

The modified method of Ben et al. [28] was used for nitrogen source preparation. Heads, viscera, bones, and muscle residues were the FB. The CB consisted of chicken lungs, intestines, and pancreas. All these nitrogen sources were aseptically collected in fish shops and poultry slaughterhouses of the market complex of IIT Guwahati (Assam, India). After cleaning, they were transported to the laboratory where they were subjected to various treatments. Fish and CB were minced by a blender, mixed with sterile water (500:1 g/1), and heated at 100°C for 20 min. After this heat pretreatment, the insoluble material was removed by centrifugation (7800 rcf/7 min), and the supernatant (FB and CB) was collected and freeze-dried for a period of 3 days. The obtained powder was kept at 4°C for further work. The production yield of powders from the different supernatants was determined as follows: The supernatant (25 mL) was collected into preweighed Falcon (50 mL), and after lyophilization, this falcon was weighed to obtain the weight of the powder.

#### 2.2.2. Determination of the Protein Content of Chicken and Fish by-product Supernatants

It was assessed according to the methods described by Bradford [29]. The supernatant of each nitrogen source was diluted 20 times. Subsequently, 1 mL of Bradford's reagent was added to 200 *μ*L of the previous dilution, and then, the test tube was vortexed and incubated at room temperature for 10 min. After incubation, the content of the tube was transferred to cuvettes, and the absorbance was measured with a spectrophotometer (Shimadzu UV-200, USA) at a wavelength of 595 nm. The amount of protein was calculated based on a calibration curve previously drawn using a standard BSA solution.

#### 2.2.3. Nitrogen Determination of the Powder Obtained From Chicken and FB After Lyophilization and That of the Yeast Extract

The total nitrogen of different samples was determined according to the Kjehdahl method as described by Jiwang and Ajay [30]. Digestion and distillation were carried out on the Total Kjehdahl Nitrogen (TKN) analyzer (Pelican, Kelplus-Distyl EM VA, India). A total of 0.2 g of the powders previously obtained after freeze-drying, the catalyst mixture (potassium sulfate: 2.5 g; cupric sulfate: 0.5 g), and 10 mL of concentrated H_2_SO_4_ were introduced into the digestion tube and placed in the TKN analyzer for 3 h at 420°C. After the digestion step, a solution of 100 mL with distilled water was prepared, and then, 10 mL of the diluted sample was taken. Twenty milliliters of 40% NaOH was added to the previous volume of sample and this mixture was kept in a distillation tube and placed in the TKN analyzer for distillation. After starting the distillation process, the tip of the condenser was immersed in a 25 mL boric acid solution (mixed with indicator) in a 250-mL conical flask. Approximately 150 mL of the distillate solution was collected and titrated with the standardized H_2_SO_4_ (0.02 N). Thus, the appearance of a purple colour indicated the end point of the titration. The TKN here expressed as a percentage is given by the following formula:

 $$\begin{array}{c} \text{TKN }\left(\%\right)=\frac{\left[\left(A-B\right)\left.\times N\times 14\right)\right]}{\text{Wt}}\times 10 \end{array}$$

where *A* indicates the milliliter of standard H_2_SO_4_ used for the sample, *B* indicates the milliliter of standard H_2_SO_4_ (or HCl) used for blank, *N* is the normality of standard H_2_SO_4_, and *Wt*. is the weight of the sample (g).

Digestion
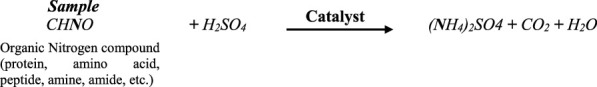


Distillation
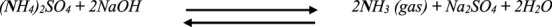


Titration
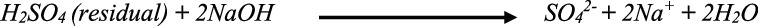


### 2.3. Preparation of a Carbon Source From Papaya Waste for Lactic Acid Production

Papaya wastes were collected from one of the Indian Institute of Technology Guwahati (IITG) canteens and sent to the laboratory for further processing. The carbon source preparation was performed based on the modified method of Pushparani et al. [31]. About 500 g of these substrates were steam treated in an autoclave at 121°C for 20 min. Once cooled, distilled water was added to the pretreated material to make a volume of 1 L and was then boiled at 80°C for 30 min in a water bath. Finally, the hydrolysate was recovered by filtration using a vacuum pump; indeed, the nylon membrane filter used had a filtration rating of 0.45 *μ*m and a diameter of 47 mm.

### 2.4. Lactic Acid Production in the Nitrogen and Carbon Source Alternative Medium

#### 2.4.1. Inoculum Preparation and Fermentation Conditions for Lactic Acid Production

The method described by Ngouénam et al. [6] was used for the inoculum preparation. The 12 h-old cultures were streaked on previously cast and solidified MRS agar and then incubated at 30°C for 48 h. Then, the bacteria were aseptically collected from the surface of the agar and suspended in 10 mL of a sterile NaCl solution (0.9%). The mixture was then vortex agitated and its opacity adjusted to Mc Farland's scale 2 (approximately 6 × 10^8^ CFU/mL). This cell suspension was used as inoculum for the fermentation.

Batch cultures were performed in 100 mL Erlenmeyer units with a useful volume of 20 mL of the supplemented and sterile carbon source. The production media (three, different in terms of nitrogen source) for the fermentation was prepared using the composition described by Ngouénam et al. [6]: hydrolysate: 1000 mL; sodium acetate: 5 g; MgSO_4_.7H_2_0: 0.6 g; MnSO_4_.H_2_0: 0.05 g; K_2_HPO_4_: 0.8 g; KH_2_PO_4_: 0.8 g; and FeSO_4_: 0.05 g. To have the same percentage of nitrogen (26, 25 g), the production mediums were supplemented with nitrogen sources as follows: yeast extract: 30 g/L; CB: 30.5 g/L; and FB 36.7 g/L; the pH of the production medium was 6.51. Then, this fermentation medium was inoculated at a rate of 10% (*v*/*v*) and incubated at 30°C under agitation (180 rpm). Substrate consumption, biomass evolution, and lactic acid production were determined on a regular time range (0, 4, 8, 12, 16, 20, and 24 h).

#### 2.4.2. Estimation of Reducing Sugars, Lactic Acid, and Biomass Evolution in the Fermentation Broth

##### 2.4.2.1. Reducing Sugar Estimation

The reducing sugars were estimated using the DNS (3, 5-dinitro salicylic acid) method described by Fisher and Stein [32].

##### 2.4.2.2. Lactic Acid Estimation

Lactic acid was quantified by the spectrophotometric method as described by Borshchevskaya et al. [33]. One (1) milliliter of the fermentation broth was centrifuged at 12,000 RCF for 5 min, and the supernatant was collected and diluted 10 times. Subsequently, 0.1 mL of the previous dilution was added to 4 mL of a FeCl_3_ solution (0.2%); after homogenization, the optical density was measured with a spectrophotometer (Thermo Scientific BioMate 3S UV-Visible spectrophotometer, Thermo Scientific, USA) at a wavelength of 390 nm. The amount of lactic acid was calculated based on a previously drawn calibration curve. The results obtained were the mean of three experiments. Lactic acid productivity (PV), lactic acid yield, and cell growth rate (*R*_*x*_) were calculated as follows:

 $$\begin{array}{c} PV\left(g/l.h\right)=\frac{\text{P}}{\text{T}}\;Rx\left(g/h\right)=\frac{\text{X}}{\text{T}}\;\;Yield\left(g/g\right)=\frac{\text{P}}{\;500} \end{array}$$

where **P** indicates the lactic acid concentration; **T** is the duration of fermentation; **X** indicates biomass; 500 is the amount of papaya by-products used for the formulation of production medium.

##### 2.4.2.3. Biomass Evolution of Fermentation Agent in the Production Medium

The biomass evolution was done by the method described by Senedese et al. [34] with slight modifications. The samples (2.0 mL) collected during fermentation were placed into preweighed Eppendorf tubes and centrifuged at 12000 RCF for 7 min at 25°C, and the supernatant was discarded. Following this, the tubes were redried at 65°C for 24 h and weighed to obtain the dry cellular weight.

### 2.5. Cost Estimation of the Formulation of Production Media

The production medium in which the highest lactic acid concentration was recorded and that containing yeast extract as a nitrogen source was evaluated for formulation cost. The components considered included sodium acetate, magnesium sulfate heptahydrate, manganese sulfate monohydrate, potassium phosphate dibasic, potassium dihydrogen phosphate, iron sulfate, CB, and papaya by-products. Considering the quantities of each component used in the formulation of 1 L of production medium, and based on the prices given by their suppliers, the costs were estimated.

### 2.6. Statistical Analysis

The quantitative data resulting from three replications were presented as mean with standard deviation using Microsoft Excel version 13 software. Then, they were analyzed by the analysis of variance (ANOVA) test using Minitab 19 software, followed by comparisons of the means between them by the Fisher test at 0.05 probability threshold.

## 3. Results and Discussion

### 3.1. Isolation and Characterization of LAB

#### 3.1.1. Isolation and Phenotypic and Biochemical Characterization of LAB

Among the 22 supposed LAB isolates obtained from overripe orange, only two isolates (NGO23 and NGO25) showed an inability to produce catalase which is an enzyme that catalyzes the reaction of oxygen formation from hydrogen peroxide [35]. This was evidenced by the absence of bubble production (effervescence) when colonies of these isolates were treated with hydrogen peroxide on a microscopic slide. Considered as isolates of interest, both these isolates were used in further work.

**Figure 2:**
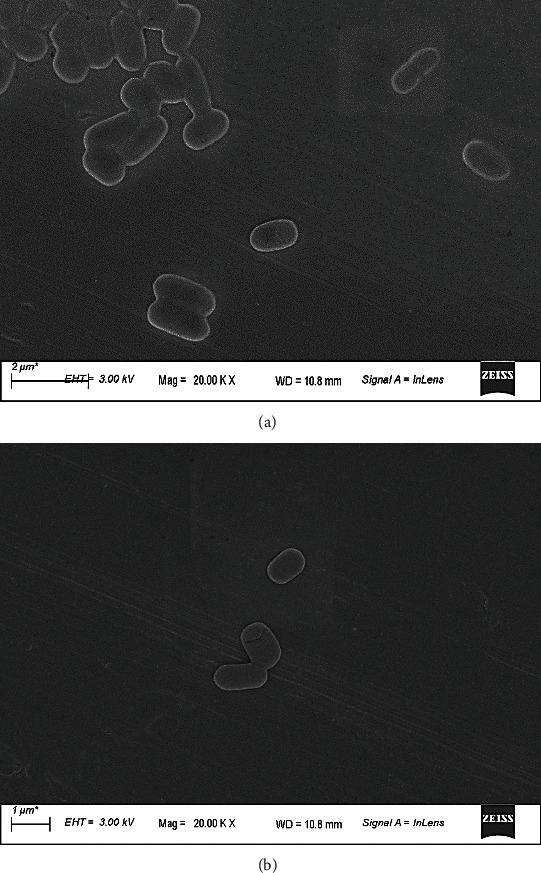
Field emission scanning electron microscope imaging of LAB isolates. (a) Isolate NGO23. (b) Isolate NGO25.

Microscopic observation of the aforementioned isolates revealed that they exhibited rod-like cell shapes with association patterns and sizes varying from one isolate to another. This rod cell shape has also been confirmed by FESEM images (Figure 2). The results of the catalase production test and microscopic observation suggest that these isolates could be LAB. However, it was essential to confirm this hypothesis by Gram staining.

Gram staining is a differential procedure that allows bacteria to be divided into two main groups: Gram-positive and Gram-negative bacteria. In the present study, the results were positive for the two isolates tested; in fact, these isolates presented a purple coloration after safranin treatment and microscopic observations.

The requirement of sodium chloride as the physiological saline prevents the cell from osmotic shock; it is an important physiological parameter for the growth of a cell [20]. In the present investigation, all LAB isolates were able to withstand salt stress up to 6.5% NaCl; however, turbidity was higher when the NaCl concentration in MRS broth was 4%. Similar findings were recorded by Ismail 2018 et al. [36].

Based on the fermentation characteristics, LABs are either homofermentative or heterofermentative. In the first case, only lactic acid is produced. In the second case, in addition to lactic acid, acetic acid, or ethanol, carbon dioxide and formic acid are produced [37]. In the context of our study, both isolates of LAB were found to be homofermentative as they were unable to produce CO_2_ from glucose. Indeed, homofermentative LABs are used as fermentation agents to maximize the yield of LA production on the one hand and to facilitate downstream processing on the other hand.

The ability of LAB isolates to hydrolyze gelatin is revealed on nutrient gelatin agar by the appearance of clear halos around the spots when the surface of the petri dish is sprayed with a saturated solution of ammonium sulfate. In this work, no clear zones were observed around the LAB spots implying that isolates NGO23 and NGO25 did not produce gelatinase.

Arginine dihydrolase is responsible for the decarboxylation of arginine to putrescine resulting in the elevation of the pH of the medium and the purple staining [38]. In this study, the arginine dihydrolase broth staining remained unchanged (purple) after 24 h of incubation implying that both LAB isolates were able to produce arginine dihydrolase. These results are in agreement with those of [38], who focused on screening and evaluation of *Lactobacillus* spp. For the development of potential probiotics, they showed that among 20 *lactobacillus* isolates of their collection, 16 were able to hydrolyse arginine.

Thakur et al. [39] in the course of their work showed that strain of *Lactobacillus delbrueckii* subsp. *bulgaricus* and *Lactiplantibacillus plantarum* did not produce urease; this was observed by no change in the urease broth coloration, which remained light yellow. Similar data were observed in our research for isolates NGO25 and NGO23 after 24 h of incubation.

Black LAB spots after 48 h of incubation, indicating a positive result for hydrogen sulfide production [23]. Within the framework of the present investigation, these observations were not recorded because both LAB isolates did not reduce the sulphur compounds to the sulfide, and therefore, they were not the combination of the sulfide compound with iron compounds to produce FeS (a black precipitate).

Under the action of the enzyme tryptophanase, tryptophan is converted into a molecule of indole, pyruvate, and ammonium [40]. The indole is highlighted in the medium by the appearance of a pink/red layer forming on top of the well when Kovac's reagent is added. Isolates NGO23 and NGO25 did not produce tryptophanase and therefore indole as the colour of the medium (colourless) remained unchanged after the addition of Kovac's reagent.

The methyl red test detects the production of acids formed during metabolism using a mixed acid fermentation pathway using pyruvate as a substrate. The pH indicator methyl red was added to wells containing isolates NGO23 and NGO25, and a red colour appears indicating a positive test.

The Voges–Proskauer test is used to highlight the presence of acetylmethylcarbinol (acetoin), an intermediate of the 2,3-butanediol fermentation pathway [41]. This metabolite was absent in the wells containing the tested isolates because, after the addition of Baritt reagents (A and B), the colour of the medium became slightly copper.

Isolates NGO23 and NGO25 showed their inability to use citrate as the sole source of carbon and energy. Indeed, the wells containing citrate kept their green coloration after 24 h of incubation, thus testifying to the non-use of citrate. The same observation has been reported by Thakur et al. [39].

LAB need a carbon source for their growth. Dhameliya et al. [42] in the course of their study showed that LAB isolated from fermented pulses were able to use some carbohydrates as carbon sources (lactose, glucose, Maltose, starch, xylose, fructose, and sucrose) and not others (glycerol, cellulose, and inositol). In the present research, this phenomenon has also been highlighted; in fact, isolates NGO25 and NGO23 were able to utilize lactose, glucose, fructose, and sucrose, while adonitol, arabinose, sorbitol, mannitol were not catabolized by both isolates. On the one hand, these observations would suggest that these LAB isolates lack genes coding for the synthesis of enzymes required for the catabolism of these compounds. On another hand, this lack of activity could be due to the total absence of expression of these different genes [43].

According to *Bergey's Manual of Systematic Bacteriology* and based on the data summarized in Table 1 as well as the aforementioned explanation concerning the phenotypic and biochemical parameters of isolates NGO25 and NGO23, it emerges that these micro-organisms are indeed LAB. In addition, their ability to grow simultaneously at 45°C and 10°C allows us to classify them in the genus *Lactobacillus* Group II [44]. The presence of LAB in oranges can be explained by their composition; carbohydrates, minerals, vitamins, and nitrogen are the nutrients found in this biotope [45]. The latter are essential elements for the growth of microorganisms, particularly LAB [12, 37, 46].

#### 3.1.2. Molecular Identification of LAB

##### 3.1.2.1. Extraction of GDNA and Gel Electrophoresis

**Figure 3:**
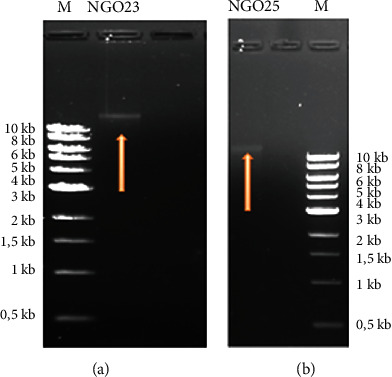
Agarose gel electrophoresis image of genomic DNA extraction of LAB isolates. (a) NGO23 isolate. (b) NGO25 isolate. The lane (M) is the DNA molecular weight ladder and the red arrow-labelled lane is the genomic DNA of the isolate in (a, b) both images.

After the extraction of the GDNA, the obtained products were submitted to gel electrophoresis which allowed us to obtain the profile below (Figure 3). On this electrophoregram, the presence of the GDNA with a molecular weight higher than 10 kb can be clearly deduced.

##### 3.1.2.2. Amplification of 16S rRNA Gene by PCR and Gel Electrophoresis

**Figure 4:**
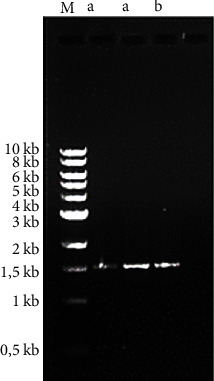
Agarose gel electrophoresis image of 16S rRNA gene after amplification by PCR. Lane (M) is the DNA molecular weight ladder, lane (a) is the NGO23 isolate, and lane (b) is the NGO25 isolate.

The amplification of the 16S rRNA gene by PCR resulted in the following profile (Figure 4) after electrophoresis. It can be highlighted from this figure that the gene coding for the 16S rRNA in all isolates was amplified and consists of approximately 1500 base pairs. These results are in agreement with those of Dhameliya et al. [42] who obtained the same size of PCR amplification products of 16S rDNA. However, the amplified nucleotide sequences of the 16S rDNA obtained in our study are higher than the one (1000 bp fragment) of the strain *Lactococcus lactis* F01 isolated from *Cyprinus carpio* by Fotso et al. [47] in the framework or their work. This difference in size could be explained by the purity of GDNA, the quality and target of the primers on the gene, and PCR conditions [48].

##### 3.1.2.3. 16S rRNA Gene Sequencing and Identification

After purification of the amplicons, they were sequenced; the sequences obtained were compared with those of the NCBI (National Centre for Biotechnology Information, Maryland, United States) GenBank database (http://blast.ncbi.nlm.nih.gov/blast.cgi). These sequences were also submitted to the NCBI GenBank for archiving and accession numbers. All these LAB isolates belong to the species *Lactiplantibacillus plantarum*, and the accession numbers of their 16S rRNA gene sequences assigned by the NCBI GenBank database were OP555442 and OP555443 for strains NGO23 and NGO25, respectively. Literature reported that the LAB of the *plantarum* species belongs to the plant microbiota hence the name *plantarum.* In addition, previous studies showed that orange was the biotope for the isolation of LAB of the genus *Lactiplantibacillus plantarum* [15, 49].

### 3.2. Powdered Nitrogen Source From Chicken and Fish By-products and Nitrogen Content

#### 3.2.1. Protein Estimation in Supernatant

The results of the estimation of the amount of protein in the supernatants prepared from fish and CB are grouped in the table below (Table 2). From this table, it can be revealed that the supernatant prepared from CB contains the highest amount (1.7251 ± 0.001 mg/mL) of protein; a value significantly higher than that of FB supernatant. The difference in protein concentration observed in these supernatants can be explained by the origin, genus, species, diet, geographical and climatic conditions, and the different parts of the animal constituting the by-products [6, 16].

#### 3.2.2. Production Yield of Powders From Different Supernatants

Table 2 also shows the production yields of the powders obtained. It appears that the highest yield was obtained with the chicken by-product supernatant (16.6 g/L). This data is explained by the high amount of protein previously obtained in the chicken by-product supernatant which was higher than the one observed in the FB supernatant.

#### 3.2.3. Total Nitrogen Content of Yeast Extract, Fish, and CB

From Table 3, it can be observed that the total nitrogen content of yeast extract, fish, and chicken by-product values varied between 71.4% DM and 87.5% DM with the highest value recorded for the yeast extract. However, no significant difference (*p* > 0.05) was observed between the value of yeast extract and the one of CB. The difference in total nitrogen of nitrogen sources can be attributed to the origin, genus, species, diet, geographical and climatic conditions, and the different parts of the animal constituting the by-products as reported by Ngouénam et al. [6] and Pambuwa and Tanganyika [16]. However, this dissimilarity can also be explained by the methods used for the preparation of the different nitrogen sources [50, 51].

### 3.3. Lactic Acid Production in the Nitrogen and Carbon Source Alternative Medium

#### 3.3.1. Kinetic of Lactic Acid Production in Different Production Media

**Figure 5:**
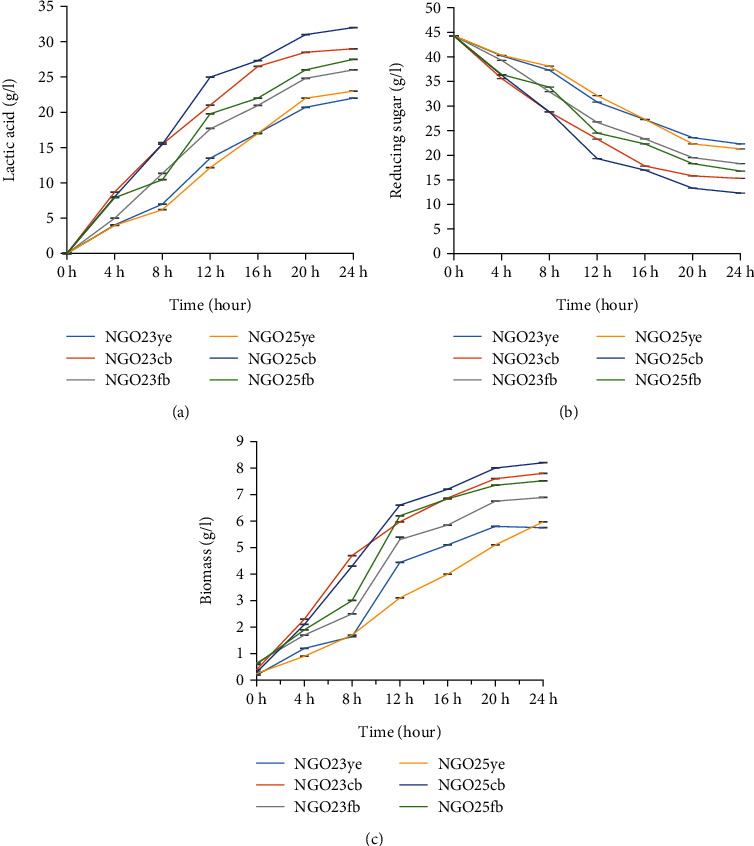
(a) Time course of LA production, (b) reducing sugar consumption, and (c) biomass evolution in different production medium. ye, production medium containing yeast extract as a nitrogen source; bc, production medium containing chicken by-products as a nitrogen source; fb, production medium containing fish by-products as nitrogen source.

Figure 5(a) shows the kinetic of LA production from *Lactiplantibacillus plantarum* NGO23 and *Lactiplantibacillus plantarum* NGO25 grown on nitrogen and carbon source alternative medium. It is clear from this figure that the LA production kinetics vary not only from one production medium to another but also from one strain to another. Similar findings have been reported by Atiat et al. [52] as part of their work on the bioproduction of lactic acid from salted whey and whey permeate. The aforementioned strains show increasing LA production between 0 and 24 h; however, LA production becomes very low after 20 h of incubation. In the present research, cultures were performed in batch mode, so the low productivity of LA after 20 h of incubation can be due to the acidity of the medium caused by the important accumulation of LA. These conditions would lead to a gradual halt in the growth and metabolism of fermentation agents, followed by their death [53]. The evolutions of the reducing sugar content in the different production media as well as the production of biomass during 24 h are represented by Figures 5(b) and 5(c), and there is an increasing production of biomass and a subsequent decrease in the concentration of reducing sugars over time. This phenomenon is explained by the fact that microorganisms need a source of carbon to ensure their growth and, therefore, the synthesis of metabolites, which, in this case, is lactic acid. As a result, an increase in biomass would lead to a subsequent decrease in the source of carbon in the production medium. These results are in agreement with those of Abdel-Rahman et al. [2] who used *Enterococcus faecium* Strain S6 as a fermentation agent for LA production from xylan-derived sugars. Due to the low pH of the production broth after 20 h of incubation, microbial growth was no longer favourable, resulting in a nonsignificant increase in biomass; similar observations have been reported by many authors [54].

#### 3.3.2. Performances of Lactic Ferments in Different Production Media

It is clearly shown in Table 4 that in the presence of CB, *Lactiplantibacillus plantarum* strain NGO25 in 24 h of fermentation produces 31.945 ± 0.078 g of LA per liter of production medium and consequently a volumetric productivity of 1.331 ± 0.003 g of LA per liter per hour. This is the highest concentration observed when the fermentation was carried out throughout 24 h. This LA concentration is significantly higher (*p* < 0.05) than those recorded in the production media containing FB (27.425 ± 0.106) and that containing yeast extract (22.975 ± 0.035 g/L) as a nitrogen source. Regarding the biomass and cell growth rate of the strain, the highest significant (*p* < 0.05) values (7.925 ± 0.035 g/L; 0.330 ± 0.001 g/L.h) were observed with strain *Lactiplantibacillus plantarum* NGO25 in the medium containing CB as nitrogen source. The strain *Lactiplantibacillus plantarum* NGO25 showed the lowest performance in the production medium containing yeast extract as a nitrogen source. The kinetic parameters of lactic acid production *Lactiplantibacillus plantarum* strain NGO23 after 24 h of fermentation are shown in Table 5. From this table, it can be seen that the highest lactic acid productivity (1.215 ± 0.009 g/L), biomass (7.405 ± 0.007 g/L), and cell growth rate (0.309 ± 0.000 g/h) were obtained in the production medium contained CB as nitrogen source. Furthermore, all these values are significantly higher (*p* < 0.05) than those recorded in the other production media. It can also be seen from this table that the lowest performance (PV: 0.921 ± 0.006 g/L.h; biomass 5.545 ± 0.021 g/L; cell growth rate 0.231 ± 0.001 g/L.h) were recorded in the production medium containing yeast extract as a nitrogen source.

The abovementioned performance (Tables 4 and 5) shows that for the same strain, the LA concentration varies from one production medium to another, and yet all the production media have the same reducing sugar content at the start of fermentation. Similar data were reported by Ngouénam et al. [6] in the framework of their investigation on the optimization of lactic acid production from pineapple by-products and an inexpensive nitrogen source using *Lactiplantibacillus plantarum* strain 4O8. This dissimilarity is probably due to the nitrogen source. Indeed, for the nitrogen requirements of the fermentation agents to be met to allow appreciable growth of the latter and consequent production of LA, the production medium must contain a sufficient quantity of protein or any other nitrogen source [55]. However, the availability and optimal use of these proteins by lactic strains require appropriate enzymatic machinery as it is clear that the proteins provided by yeast extract, fish by-products, and CB are different from each other [56, 57]. Thus, the fermentation agent that has the appropriate proteases will find it easy to obtain the amino acids it needs as a nitrogen source, which will enable it to ensure its metabolism and produce the molecule of interest [58, 59]. Similarly, the richness of the production medium in certain amino acids and adequate proportions would have a positive impact on the yield of LA production. Srivastava et al. [60] in the course of their work showed that a combination of L-phenylalanine and L-lysine (34.0 mg/L) gave a maximum lactic acid yield of 64.86 ± 0.2 g/L. Another parameter that needs to be taken into account is the presence of vitamins in these by-products. Vitamins are growth factors; they promote the development of micro-organisms, particularly LAB. In addition, their presence, especially Group B vitamins [46, 61], in different concentrations in the production media, could also explain the differences in kinetic parameters observed for the same strain in the different production broths. The present study shows that the highest performance by the two fermentation agents was in the medium containing CB as a nitrogen source. However, the performance of *Lactiplantibacillus plantarum* strain NGO25 (VP: 1.331 ± 0.003 g/L.h) remains significantly higher (*p* < 0.05) than that of *Lactiplantibacillus plantarum* NGO23 (VP: 1.215 ± 0.009 g/L.h). In addition, the CB were the source of nitrogen that allowed a better growth of lactic ferments, and consequently, the lactic acid production, although no significant difference (*p* > 0.05), was previously observed between the total nitrogen content of the latter this nitrogen source and that of yeast extract. The use of papaya by-products and yeast extract as substrates for lactic acid production in batch mode using *Lactiplantibacillus plantarum* strain 4O8 as the fermentation agent was investigated by Ngouénam et al. [15]. Under these conditions, these authors obtained a LA concentration of 25.81 g/L which is significantly lower than the best acidifying activity activity (31.945 g/L) recorded by *Lactiplantibacillus plantarum* strain NGO25 in the present research using the same carbon source and freeze-dried CB as nitrogen source. Similarly, the highest lactic acid productivity (1 g/L.h) highlighted by Atiat et al. [53] within the framework of their work on the use of salted whey and whey permeate for lactic acid production by a batch culture using *Lacticaseibacillus casei* is lower than the obtained value (1.331 g/L.h) in the present research, although their production medium contained yeast extract as nitrogen source. In the same line, Selim et al. [59] in their work recorded an LA volumetric productivity of 0.93 g/L.h and this batch mode; in addition, the fermentation agent was *Enterococcus faecium* Strain WH51-1, and the carbon and nitrogen sources were corn steep water and yeast extract, respectively. In a production medium containing *Caldicellulosiruptor* sp. strain DIB 104C as a fermentation agent, microcrystalline cellulose and yeast extract as substrates, the lactic acid productivity of 1 g/L.h was highlighted under batch condition [62]. The latter activity is lower than the one obtained in the present research. However, the literature also provides better data than those obtained in our work, including a volumetric productivity of 13.5, 1.57, 18.56, 3.74, 3.37, and 2.426 obtained, respectively, by Abdel-Rahman [2], Gonzalez et al. [63], Ma et al. [64], Oliveira et al. [65], Beitel et al. [66], and Jianfei et al. [67] in their research. It is important to note that in the aforementioned investigations, the carbon and nitrogen sources were xylose, yeast extract, peptone, beef extract, corn steep liquor, sorghum hydrolyzates, rice straw, sucrose, sugarcane molasses, and fructose. Based on these observations, many plausible explanations could be at the origin of the dissimilarity in terms of lactic acid production noted in our study and the data of the literature; this includes the chemical composition of carbon and nitrogen substrates as well as the methods used for their preparation, the nature of the fermentation agent, the fermentation conditions, the production medium composition, and fermentation duration [60–67].

### 3.4. Cost Estimation for the Formulation of Production Media

In order to have a clear idea about the possibility that freeze-dried CB could be a real alternative to yeast extract in the production of lactic acid, the formulation costs of production media containing these different nitrogen sources were evaluated and the results obtained are shown in Table 6. From this table, it appears that the formulation cost of the production medium containing yeast extract is 11.13 times ($1.692) higher than that containing the alternative nitrogen source ($0.152). These results are in agreement with the literature, which reports that the use of yeast extract is among the main problem of the production cost of LA by fermentative pathway [6–8].

## 4. Conclusion

In summary, the strains *Lactiplantibacillus plantarum* NGO25 and NGO23 isolated from oranges can use fish and CB as an alternative to yeast extract and papaya by-products as carbon sources in the production of LA. Indeed, the highest LA concentrations were recorded by the two fermentation agents in the chicken by-product medium. Furthermore, the best kinetic parameters of LA production (lactic acid (g/L): 31.945 ± 0.078; volumetric productivity (g/L.h): 1.331 ± 0.003; lactic acid yield (mg/g) 6389 ± 0.156; biomass (g/L) 7.925 ± 0.035; and cell growth rate (g/L.h): 0.330 ± 0.001) were recorded by *Lactiplantibacillus plantarum* NGO25 after 24 h of fermentation in the CB medium. In addition, this production medium was 11.13 times less expensive to formulate than that containing yeast extract as a nitrogen source. Hence, CB could be a solution to the high LA production costs linked to the nitrogen source. However, the knowledge of the amino acid profile of these inexpensive nitrogen sources as well as their vitamin contents would be useful for their large-scale utilization in LA production.

## Figures and Tables

**Table 1:** Phenotypic and biochemical parameters of LAB isolates.

**Parameters**	**NGO23**	**NGO25**
Microscopic observation	Rod shape	Rod shape
Gram staining	***+***	***+***
Catalase production	***−***	***−***
NaCl tolerance (2, 4, 6.5%),	***+***	***+***
CO_2_ production	***−***	***−***
Growth at 10°C and 45°C	***+***	***+***
Indole test	***−***	***−***
Methyl red test	***+***	***+***
Voges–Proskauer test	***−***	***−***
Arginine hydrolysis	***+***	***+***
Gelatine hydrolysis	***−***	***−***
Urease production	***−***	***−***
Hydrogen sulfide production	***−***	***−***
Citrate utilization	***−***	***−***
Glucose	***+***	***+***
Adonitol	***−***	***−***
Arabinose	***−***	***−***
Lactose	***+***	***+***
Sorbitol	***−***	***−***
Mannitol	***−***	***−***
Rhamnose	***−***	***−***
Sucrose	***+***	***+***
Fructose	***+***	***+***
Genus	*Lactobacillus* Group II	*Lactobacillus* Group II

**Table 2:** Protein content of by-product supernatant and production yield of powders from different supernatants.

**Sample**	**Protein concentration (mg/**mL**)**	**Sample (L)**	**Production yields of the powders (g)**
Chicken by-products supernatant	1.7251 ± 0.001^a^	Chicken by-products supernatant	16.6
Fish by-products supernatant	1.275 ± 0.000^b^	Fish by-products supernatant	12.933

^a,b^The values with different letters on the same column differ significantly from each other (*p* < 0.05).

**Table 3:** Total nitrogen content of nitrogen sources.

**Sample**	**Fish by-products**	**Chicken by-products**	**Yeast extract**
Total nitrogen (%DM)	71.4 ± 0.000^b^	86.145 ± 0.001^a^	87.5 ± 0.99^a^

^a,b^The values with different letters on the same line differ significantly from each other (*p* < 0.05).

**Table 4:** Kinetic parameters of lactic acid production of *Lactiplantibacillus plantarum* NGO25 after 24 h of fermentation.

**Parameters**	**PMYE**	**PMCB**	**PMFB**
Lactic acid (g/L)	22.975 ± 0.035^c^	31.945 ± 0.078^a^	27.425 ± 0.106^b^
VP (g/L.h)	0.957 ± 0.001^c^	1.331 ± 0.003^a^	1.143 ± 0.004^b^
Lactic acid yield (mg/g)	45.95 ± 0.071^c^	63.89 ± 0.156^a^	54.85 ± 0.212^b^
*X* (g/L)	5.705 ± 0.021^c^	7.925 ± 0.035^a^	6.905 ± 0.021^b^
*R*_*x*_ (g/L.h)	0.238 ± 0.001^c^	0.330 ± 0.001^a^	0.287 ± 0.002^b^

^a,b,c^The values with different letters on the same line differ significantly from each other (*p* < 0.05).

Abbreviations: PMBC, production medium containing chicken by-products as nitrogen source; PMBF, production medium containing fish by-products as nitrogen source; PMYE, production medium containing yeast extract as nitrogen source; *R*_*x*_, cell growth rate; VP, volumetric productivity; *X*, biomass.

**Table 5:** Kinetic parameters of lactic acid production of *Lactiplantibacillus plantarum* strain NGO23 after 24 h of fermentation.

**Parameters**	**PMYE**	**PMCB**	**PMFB**
Lactic acid (g/L)	22.1 ± 0.141^c^	29.15 ± 0.212^a^	26.25 ± 0.354^b^
VP (g/L.h)	0.921 ± 0.006^c^	1.215 ± 0.009^a^	1.094 ± 0.015^b^
Lactic acid yield (mg/g)	44.2 ± 0.283^c^	58.3 ± 0.4242^a^	52.5 ± 0.707^b^
X (g/L)	5.545 ± 0.021^c^	7.405 ± 0.007^a^	6.23 ± 0.0141^b^
R_x_ (g/L.h)	0.231 ± 0.001*c*	0.309 ± 0.000^a^	0.259 ± 0.001^b^

^a,b,c^The values with different letters on the same line differ significantly each other (p <0.05).

Abbreviations: PMBC, production medium containing chicken by-products as nitrogen source; PMBF, production medium containing fish by-products as nitrogen source; PMYE, production medium containing yeast extract as nitrogen source; *R*_*x*_, cell growth rate; VP, volumetric productivity; *X*, biomass.

**Table 6:** Cost price for the formulation of 1 L of production medium.

**Components**	**Cost per 500 g ($)**	**Source supplier**	**PMCB**		**PMYE**	
			**Amount used (g)**	**Cost ($)**	**Amount used (g)**	**Cost ($)**
Sodium acetate (C_2_H_3_NaO_2_)	9.28	Himedia	5	0.093	5	0.093
Magnesium sulfate heptahydrate (MgSO_4_.7H_2_0)	6.64	Merck	0.6	0.008	0.6	0.008
Manganese sulfate monohydrate (MnSO_4_.H_2_0)	98.77	Sigma	0.05	0.01	0.05	0.01
Potassium phosphate dibasic (K_2_HPO_4)_	15.3	Himedia	0.8	0.024	0.8	0.024
Potassium dihydrogen phosphate (KH_2_PO_4_)	9.78	Himedia	0.8	0.016	0.8	0.016
Iron sulfate (FeSO_4_)	5.91	Himedia	0.05	0.001	0.05	0.001
Papaya by-products (carbon source)	0	Canteens (IITG)	500	0	0	0
Chicken by-products (nitrogen source)	0	Poultry slaughterhouses (market complex IITG)	30.5	0	∕	**∕**
Yeast extract (nitrogen source)	25.66	Himedia	∕	∕	30	1.54
Total cost for 1 L of production medium				0.152		1.692
Increase coefficient				X		11.13X

Abbreviations: $, American dollars; PMBC, production medium containing chicken by-products as nitrogen source; PMYE, production medium containing yeast extract as nitrogen source.

## Data Availability

All data will be made available on request.
